# Experimental and theoretical investigation of the mechanisms of drying during CO_2_ injection into saline reservoirs

**DOI:** 10.1038/s41598-023-36419-3

**Published:** 2023-06-06

**Authors:** Yen Adams Sokama-Neuyam, Muhammad Aslam Md Yusof, Shadrack Kofi Owusu, Victor Darkwah-Owusu, Joshua Nsiah Turkson, Adwoa Sampongmaa Otchere, Jann Rune Ursin

**Affiliations:** 1 Department of Petroleum Engineering, Kwame Nkrumah University of Science and Technology, PMB Kumasi, Kumasi, Ghana; 2 Department of Petroleum Engineering, Universiti Teknologi PETRONAS, Seri Iskandar, 32610 Perak, Malaysia; 3 Department of Energy and Petroleum Engineering, University of Stavanger, 4036 Stavanger, Norway

**Keywords:** Carbon capture and storage, Climate-change mitigation

## Abstract

A viable CO_2_ storage resource must have sufficient storage capacity, reliable containment efficiency and adequate well injectivity. Deep saline formations stand out in terms of storage capacity and containment efficiency. However, formation brine dry-out and salt precipitation in the near well region could impair CO_2_ injectivity in deep saline reservoirs, thus reducing their potential for CO_2_ storage. Core-flood experiments and analytical modelling were used to investigate various mechanisms of external and internal salt precipitation. Particularly, the impact of the extension of the dry-out region on CO_2_ injectivity was investigated. It was found that, for high permeability rocks, injection of CO_2_ at relatively low injection rates could result in salt cake deposition at the injection inlet especially under high salinity conditions. It was also found that extension of the dry-out region does not have significant impact on CO_2_ injectivity. Although the magnitude of CO_2_ injectivity impairment increased more than two-fold when initial brine salinity was doubled, real-time changes in CO_2_ injectivity during the drying process was found to be independent of initial brine salinity. We have shown that the *bundle-of-tubes* model could provide useful insight into the process of brine vaporization and salt deposition in the dry-out region during CO_2_ injection. This work provides vital understanding of the effect of salt precipitation on CO_2_ injectivity.

## Introduction

The prerequisites for successful Carbon Capture, Utilization and Storage (CCUS) are robust containment efficiency, adequate storage volume and sufficient well injectivity, to inject large quantities of CO_2_ at practical flow rates^1^. Deep saline formations are suitable storage resources for CCUS based on their storage capacity and containment^2–7^. However, salt precipitation due to brine vaporization, especially in the vicinity of the wellbore, during CO_2_ injection could affect CO_2_ injectivity in deep saline formations^8–13^. The underlying factors of CO_2_ injectivity impairment induced by salt precipitation have been extensively studied and identified to include the concentration of salt in the brine, the petrophysical and petrographic properties of the rock, the drying rate, the extent of the dry-out zone, solid salt saturation in the pore spaces after drying, distribution of precipitated salts within the pores and the petrophysical properties of the reservoir rock^14,15^.

Salt precipitation or scaling has been a major formation damage challenge in oilfield operations since the inception of the industry. In field operations involving injection, storage and production of natural gas; various levels of injectivity impairment related directly and indirectly to salt precipitation have been encountered and reported^16–19^. Permeability impairment ranging between 13 and 83% and porosity reduction around 2–15% have been reported from laboratory experiments^9,11,12,20–24^. Rigorous theoretical simulations have also confirmed the reported experimental and field findings^23,25–30^. Cui et al., (2023) have compiled a more recent update of injectivity impairment induced by salt precipitation that have been reported by various researchers through experimental and modelling studies. Generally, porosity impairment has been lower than permeability changes as the deposition of salt in the flow pathways have more impact on permeability than porosity.

During injection of CO_2_ into brine-filled rock, the injected gas initially displaces mobile brine out of the rock. During this immiscible displacement stage, mass transfer between the gas and the displaced aqueous phase is minimal. After the mobile brine has been displaced, continuous injection of CO_2_, especially under typical field injection conditions, results in vaporization of brine, drying and salt precipitation. Generally, the dry-out zone extends into the formation with injection time after the onset of the drying process. Some experimental and numerical studies have in part examined mechanisms underlying the development of the dry-out zone qualitatively and quantitatively^9,25,27,31–35^. However, to the best of knowledge of the authors, there has not been an experimental or modelling studies that has attempted to monitor the extension of the dry-out zone in real-time and examine its impact on injectivity impairment.

Miri and Hellevang^14^ have identified the main underlying mechanisms that govern the drying rate and extension of the dry-out zone during salt precipitation. These factors include: (1) immiscible two-phase displacement of the resident brine by injected CO_2_, (2) vaporization of brine into the flowing CO_2_ stream, (3) capillary-driven back-flow of brine towards the injection inlet, (4) diffusion of dissolved salt in the aqueous phase, (5) gravity override of injected CO_2_, and (6) salt self-enhancing. These factors have also been confirmed under practical field injection conditions^36^. It has also been reported that very low brine evaporation rate in the drying front, may result in homogeneous distribution of precipitated salt throughout the porous medium^9,10,14,37^. For high vaporization rates, there is no sufficient time for the salt concentration gradient to diffuse away from the drying front, resulting in nonhomogeneous accumulation of salt^10,38^. The position where salt accumulation is maximum is still largely debatable. Numerical experiments conducted by Roels et al.^39^ suggests that precipitated salt may accumulate far from the wellbore. However, other research works^6,7,9,17,22,40^ report that precipitated salt accumulates near the wellbore where the fluxes and brine vaporization are the highest. However, Berntsen et al.^41^ identified three different drying regimes in different wellbore regions when they investigated drying and salt clogging under near-realistic radial CO_2_ flow conditions. This suggests that the distribution of precipitated salt is not uniform across the drying region although the exact relative distribution of precipitated salt and the governing factors are yet to be thoroughly investigated. A detailed review and assessment of CO_2_ injectivity impairment mechanisms has been presented by Hajiabadi et al.^42^.

Several analytical and numerical models have been developed to study the physics behind salt precipitation at the core and field scales^27,28,43–45^. More recently, machine learning-based modelling has also been adopted to study the mechanisms of CO_2_ injectivity^46–48^. The microstructure of natural porous media are very complex with tortuousness and often noncircular pore channels^49–51^. Three pore-scale models have been widely used in attempt to reconstruct representative analogs of porous structure to study fluid flow in porous media; the network models, the sphere-pack model, and the *bundle-of-tubes* model^52^. The simplest pore-scale model is the *bundle-of-tubes* model which is normally derived from the Hagen–Poiseuille equation^53^. Early traditional *bundle-of-tubes* models represent porous media as an assemblage of independent, uniform circular capillary tubes^54,55^. *Bundle-of-tubes* models may be interacting or non-interacting, depending on fluid communication between the individual tubes^56^.

In the present work, we have conducted core-flood experiments using sandstone core plugs to investigate the mechanisms of internal and external salt deposition during CO_2_ injection into deep saline reservoirs. Particularly, we have investigated the development of the dry-out zone in near-real-time and attempted to monitor and quantify the impact of the extension of the drying region on CO_2_ injectivity. We then derived a tractable *bundle-of-tubes* model describing the experimental observations, in attempts to model the relationship between CO_2_ injectivity impairment and the extent of the dry-out zone, i.e.to establish a quantitative relationship between the development of the dry-out zone and CO_2_ injectivity. The results have been discussed in light of established literature.

## Experimental work

### Rock and fluids

Outcrop Berea sandstone core plugs considered to be homogeneous with brine permeability in the range of 60–100 mD and porosity between 18 and 20% were used as the main reservoir rock for the study. Each core sample has a length of 20 cm and 3.81 cm diameter. These long core plugs were selected purposely to increase the residence time of CO_2_ during the drying process.

Liquefied CO_2_ with percentage purity of about 99.7%, was used as the non-wetting phase. During liquid CO_2_ flooding, the fluid was injected at 80 bar and 25 °C. For supercritical CO_2_ flooding, the injection conditions were set to 80 bar and 45 °C.

NaCl brines, salinity of 75 g/l and 150 g/l, were selected to represent low salinity (LS) and high salinity (HS) formation brines, respectively. The HS brine is expected to represent deep saline formation brine, while the LS brine was selected to test the sensitivity of brine salinity.

### The experimental setup and procedure

**Figure 1:**
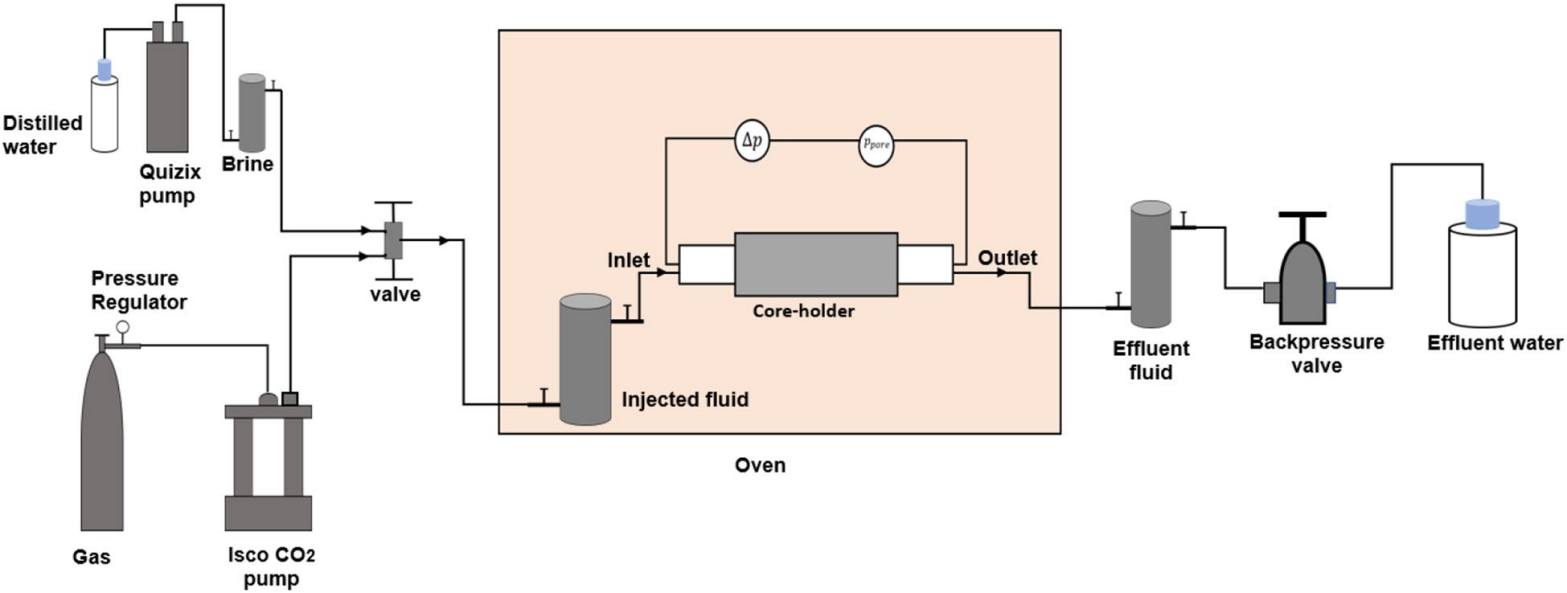
The experimental set up used for the CO_2_ core-flooding experiments.

Figure 1 depicts schematics of the core-flooding apparatus used in the experimental work. Initially, the core was loaded into the core-holder. The Quizix pump is used to deliver brine through the connected piston cell for temporary aging to attain a set uniform temperature before it arrives at the core inlet. The ISCO CO_2_ pump receives liquid CO_2_ from the gas bottle through a pressure regulator. Either liquid or supercritical CO_2_ can be injected into the core depending on the set conditions. The injected fluid passes the piston cell which holds the fluid until a preset temperature is attained in the oven. A differential pressure gauge and a pressure transducer are used to monitor the pressure drop across the core and record the pore pressure. A backpressure of 80 bar was set at the outlet, during CO_2_ injection and the effluent fluid was safely collected in a piston cell for analysis and safe disposal.

The core sample was first cleaned and dried at 65 °C for about 24 h. The core was then wrapped in shrinking Teflon sleeve before it was inserted into a rubber sleeve to prevent CO_2_ leakage. The carefully wrapped core sample was then mounted into the core holder. A confining pressure of about 150 bar was applied to the core in the core holder. The experimental procedure consists of the following:

1. Initial liquid CO_2_ permeability ($${K}_{i})$$ of the core sample was measured.
2. The core was vacuum-saturated with a specific salinity brine (HS or LS).
3. The saturated core sample was flooded with specific pore volumes (PV) of supercritical CO_2_ to vaporize brine and dry the core starting at the injection inlet.
4. The core was taken out, inspected, and the extent of the dry-out zone measured.
5. Final liquid CO_2_ permeability (${K}_{f}$) of the core was the measured.

In Step 2, the vacuum-saturated core was prepared and inserted into the core-holder followed by brine injection at 1 ml/min until complete saturation was attained. In Step 3, supercritical CO_2_ was injected into the saturated core at 5 ml/min to vaporize brine and possibly precipitate salt into the pores. After each round of supercritical CO_2_ injection, the core was inspected, and the extent of the dry-out zone was measured by scanning. In Step 1 and Step 5, liquid CO_2_ was injected at 2 ml/min to measure permeability before and after brine vaporization, and salt precipitation.

### Estimation of injectivity impairment

Pressure drop profiles and pore-pressure were recorded during CO_2_ flooding to qualitatively study the drying process and the impact of the drying on injectivity. Pressure drop profiles might not provide thorough information of pore level events, but they give useful insight into real time changes in flow properties in the core during the drying process.

For constant injection rates (${q}_{i}={q}_{f}$) under linear flow conditions in a homogenous rock, we may define the relative injectivity change index, $\beta$ from Darcy law as:

1 $$\begin{array}{c}\beta =1-\left(\frac{{\Delta p}_{i}}{{\Delta p}_{f}}\right)=1-\left(\frac{{k}_{f}}{{k}_{i}}\right)\end{array}$$

The terms $\left(\frac{{\Delta p}_{i}}{{\Delta p}_{f}}\right)$ and $\left(\frac{{K}_{f}}{{K}_{i}}\right)$ are evaluated and compared for consistency. The $\beta$ provides an indirect estimation of CO_2_ injectivity induced by the drying process. Salt accumulation in the pore constrictions will increase the pressure drop, thus decreasing the rock permeability and CO_2_ injectivity.

## Modelling work

### Objectives and underlying assumptions of the model

The main objective of the modelling work is to develop a tractable physical analytical model with just enough capability to complement the experimental studies in terms of estimating injectivity impairment induced after salt precipitation without sacrificing the effectiveness of the model to capture the major underlying mechanisms. The bundle of tubes model was selected because of its ability to capture the main physical processes of transport in porous media, its computational efficiency and flexibility for modelling different pore-scale events. The main assumptions of the model include:

1. A fully homogeneous reservoir rock
2. No chemical interaction between the injection fluid and the contents of the reservoir rock
3. Single phase flow of injected CO_2_ in the reservoir during injectivity tests
4. Uniform deposition of precipitated salt in the dry-out region of the rock

Although the model is limited by these assumptions, it captures the basic mechanisms and therefore capable of providing acceptable estimates of the quantitative impact of salt deposition on CO_2_ storage in deep saline reservoirs at the core-scale.

### Conceptualization of the model

**Figure 2:**
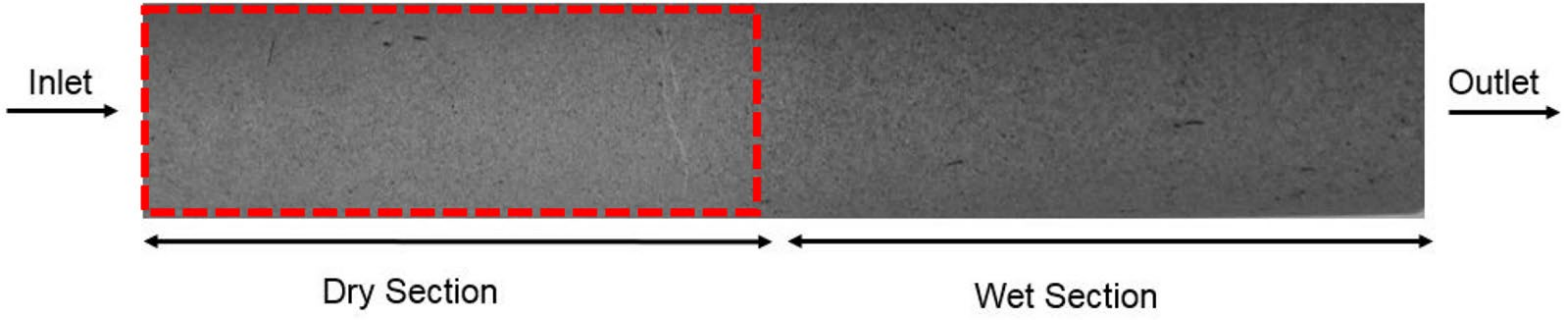
A Berea sandstone core after a period of drying. The core was initially saturated with LS brine after which it was flooded with about 160 PV of supercritical CO_2_ at injection flow rate of 5 ml/min.

Figure 2 shows a Berea core that was initially saturated with LS brine and flooded with about 160 PV of supercritical CO_2_ at an injection flow rate of 5 ml/min. It can be observed that the part of the core close to the injection inlet dried out completely after brine vaporization while the remaining section close to the outlet remained wet with immobile brine.

**Figure 3:**
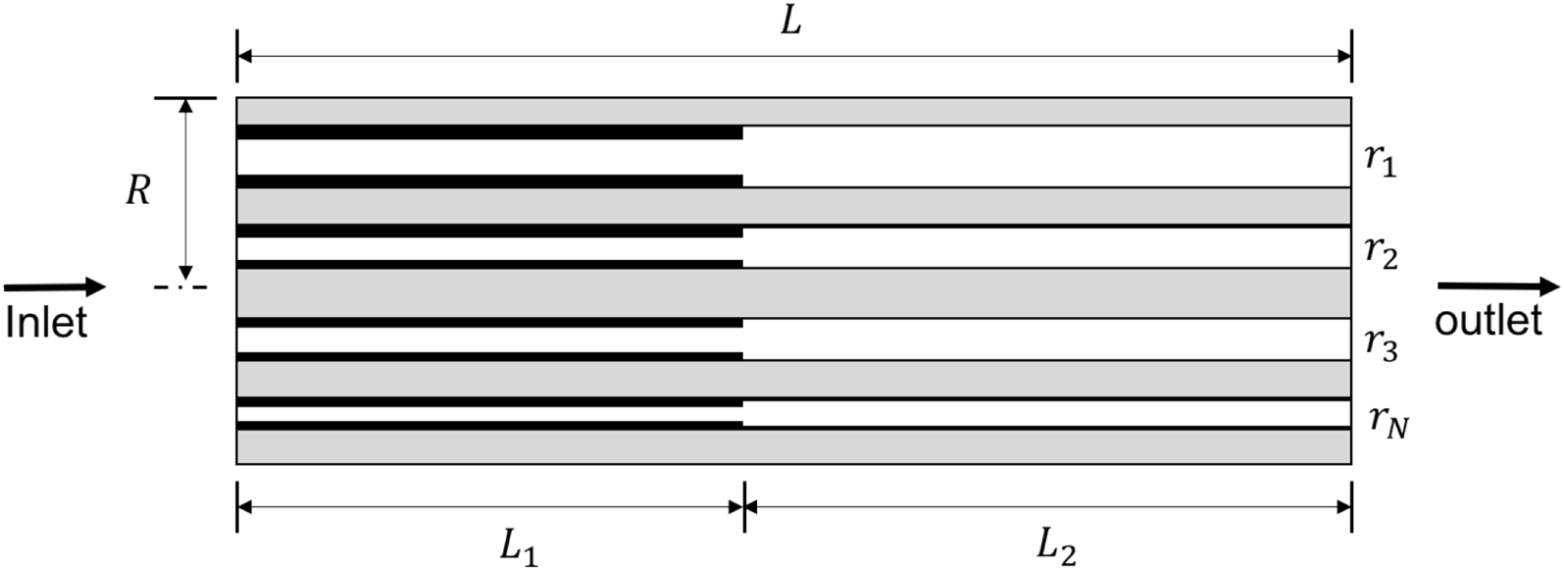
A Schematic of the bundle-of-tubes model. The core of length L and radius R is represented by a bundle of parallel cylindrical capillary tubes with varying radii interspersed between non-porous mass.

By inspection, the core can be sectioned into a dry-out region and a wet region after brine vaporization and drying (Fig. 2). Salt precipitation is expected in the dry-out region after brine vaporization. The cylindrical core, radius $R$ and length $L$ was reconstructed with a bundle of parallel cylindrical capillary tubes with varying radii (${r}_{1},{r}_{2},{r}_{3},\dots {r}_{N}$) interspersed between a non-porous mass (shaded regions) which represent the rock matrix (Fig. 3). The model is sectioned into a dry-out region (${L}_{1}$) where salt has been precipitated into the tubes and the wet region (${L}_{2}$) where the pores contain immobile brine.

In the dry-out region, the precipitated solid salt is assumed to accumulate on the walls of the pore constriction, represented by the capillary tubes. As a result, the accumulated salt in the dry-out region would reduce the flow area of the tube $r$ by $\Delta r$, for a total of $N$ capillary tubes in the porous medium.

### Modelling of injectivity impairment

We define a dimensionless parameter ${l}_{d},$ to monitor the extension of the dry-out region from the inlet to the outlet of the core of total length $L$ given by:

2 $$\begin{array}{c}{l}_{d}=\frac{{L}_{1}}{L}\quad {l}_{d}\in \left[\mathrm{0,1}\right]\end{array}$$

Using the Hagen − Poiseuille equation, the fluid injectivity through the bundle of capillary tubes can be expressed as:

3 $$\begin{array}{c}I=\frac{Q}{\Delta p}= \frac{\pi }{8\mu L}{\sum }_{i=1}^{N}\left[\frac{{\left({r}_{i}-{\Delta r}_{i}\right)}^{4}}{{l}_{d}+\left(1-{l}_{d}\right){\left(1-\frac{{\Delta r}_{i}}{{r}_{i}}\right)}^{4}}\right] \end{array}$$

In Eq. (3), $\mu$ is the viscosity of the injected fluid, $Q$ is the injection flow rate and $\Delta p$ is the total pressure drop across the bundle of tubes. The relative injectivity change index $\beta$ can then be derived as:

4 $$\begin{array}{c}\beta =1-\frac{{I}_{f}}{{I}_{i}}=1-\frac{{\sum }_{i=1}^{N}\left[\frac{{\left({r}_{i}-{\Delta r}_{i}\right)}^{4}}{{l}_{d}+\left(1-{l}_{d}\right){\left(1-\frac{{\Delta r}_{i}}{{r}_{i}}\right)}^{4}}\right]}{{\sum }_{i=1}^{N}{r}_{i}^{4}}\end{array}$$

In Eq. (4), ${I}_{i}$ and ${I}_{f}$ are the fluid injectivity before and after drying and salt precipitation, respectively. If the pore size distribution of the rock is known, Eq. (4) can be used to estimate the impact of precipitated salt on injectivity if ${l}_{d}$ and ${\Delta r}_{i}$ for each ${r}_{i}$ of the total $N$ capillary tubes are known. These are the three unknown parameters in the equation that must be determined.

The pore volume of the core is approximately equal to the sum of the internal volume of all the capillary tubes. Using the definition of porosity,$\phi ,$ the total number of capillary tubes that is representative of the pore volume of the core can be related to the porosity, the size of the core $R$ and the average pore radius, $\overline{{r }_{i}}$ by:

5 $$\begin{array}{c}N\approx \phi \frac{{R}^{2}}{\overline{{r }_{i}^{2}}}\end{array}$$

Integrating ${r}_{i}^{2}$ from zero to the maximum salt thickness ${\Delta r}_{max}$ gives:

6 $$\begin{array}{c}\overline{{r }_{i}^{2}}=\frac{1}{\Delta {r}_{i}}{\int }_{0}^{\Delta {r}_{i}}{r}_{i}^{2}d{r}_{i}=\frac{{ \left(\Delta {r}_{i}\right)}^{3}}{3}\end{array}$$

Similarly, integrating ${r}_{i}$ over the same salt thickness yields:

7 $$\begin{array}{c}\overline{{r }_{i}}=\frac{1}{\Delta {r}_{i}}{\int }_{0}^{\Delta {r}_{i}}{r}_{i}d{r}_{i}=\frac{\Delta {r}_{i}}{2}\end{array}$$

Combining Eqs. (6) and (7) gives a relationship between $\overline{{r }_{i}^{2}}$ and $\overline{{r }_{i}}$:

8 $$\begin{array}{c}\overline{{r }_{i}^{2}}=\frac{4}{3}{\overline{{r }_{i}}}^{2}\end{array}$$

where ${\overline{r} }_{i}^{2}$ is the square of the average of the capillary tube radii. Substituting Eq. (8) into Eq. (5), the total number of capillary tubes that will give a specific porosity can be estimated:

9 $$\begin{array}{c}N\approx \frac{3}{4}\phi {\left(\frac{R}{\overline{{r }_{i}}}\right)}^{2}\end{array}$$

From Eq. (9), the total number of capillary tubes that represents the pore volume of the core quantitatively can be estimated, given the porosity $\phi$ and average pore size, $\overline{{r }_{i}}$, given the pore size distribution of the rock.

The solid salt saturation ${S}_{si}$ deposited in a capillary tube is defined by:

10 $$\begin{array}{c}{S}_{si}=\frac{{V}_{si}}{{V}_{pi}}=\frac{2{l}_{d}\Delta {r}_{i}{r}_{e}}{{r}_{i}^{2}}\end{array}$$

where ${V}_{si}$ is the volume of solid salt in the tube,${V}_{pi}$ is the volume available in the tube for salt deposition and ${r}_{e}=({r}_{i}-{\Delta r}_{i}$). Substituting for ${r}_{e}=({r}_{i}-{\Delta r}_{i}$) gives:

11 $$\begin{array}{c}{S}_{si}=2{l}_{d}\Delta {r}_{i}\left(\frac{1}{{r}_{i}}-\frac{\Delta {r}_{i}}{{r}_{i}^{2}}\right)\end{array}$$

In Eq. (11), assuming ${r}_{i}\gg {\Delta r}_{i}$ that $\frac{\Delta {r}_{i}}{{r}_{i}^{2}}=0$ we can derive ${\Delta r}_{i}$ in terms of ${S}_{si}$:

12 $$\begin{array}{c}{\Delta r}_{i}=\frac{{S}_{si}{r}_{i}}{2{l}_{d}}\end{array}$$

The total mass of solid salt deposited in all the $N$ tubes can be estimated as:

13 $$\begin{array}{c}{m}_{t}=2\pi N{\rho }_{s}L{l}_{d}2\overline{{r }_{i}}\overline{{\Delta r }_{i}}\end{array}$$

where ${\rho }_{s}$ is the density of solid salt. But the total mass of salt deposited in the core can also be expressed as:

14 $$\begin{array}{c}{m}_{t}={\rho }_{s}{V}_{st}=\pi {\rho }_{s}{S}_{s}{R}^{2}L\phi \end{array}$$

where ${V}_{st}$ is the total volume of salt deposited in the core. Combining Eqs. (13) and (14) gives:

15 $$\begin{array}{c}\overline{{\Delta r }_{i}}=\frac{{S}_{s}\phi {R}^{2}}{2N{l}_{d}\overline{{r }_{i}}}\end{array}$$

Substituting Eqs. (9) into (15) gives:

16 $$\begin{array}{c}\overline{{\Delta r }_{i}}\approx \frac{2}{3}\frac{{S}_{s}\overline{{r }_{i}}}{{l}_{d}}\end{array}$$

Since ${S}_{s}$ and ${l}_{d}$ are constant at any point in time, the uncertainty in $\overline{{\Delta r }_{i}}$ in Eq. (16) is mainly associated with uncertainty in $\overline{{r }_{i}}$. Therefore, Eq. (16) can be used to estimate the average thickness of the deposited solid salt on the pore walls in terms of the cumulative solid salt saturation, ${S}_{s}$ by:

17 $$\begin{array}{c}{\Delta r}_{i}=\frac{2}{3}\frac{{S}_{s}{r}_{i}}{{l}_{d}}\end{array}$$

If ${S}_{s}$ and ${l}_{d}$ are known, we can compute ${\Delta r}_{i}$ for each ${r}_{i}$ after salt precipitation. Using mass balance, Pruess^27^ derived an equation to estimate the solid salt saturation as:

18 $$\begin{array}{c}{S}_{s}=\left(1-{\overline{S} }_{g,d}\right)\frac{{\rho }_{aq}{X}_{s}}{{\rho }_{s}}\end{array}$$

In Eq. (18), ${\overline{S} }_{g,d}$ is the average gas saturation behind the dry-out front, ${\rho }_{aq}$ is the density of brine, ${X}_{s}$ is the mass fraction of salt in the brine and ${\rho }_{s}$ is the density of solid salt. Since ${\overline{S} }_{g,d}$ is also a measure of the extension of the dry-out region similar to ${l}_{d}$ in the current model, a correlation was derived for the solid salt saturation, by fitting experimental data:

19 $$\begin{array}{c}{S}_{s}=\left(0.85+0.1{l}_{d}\right)\frac{{\rho }_{aq}{X}_{s}}{{\rho }_{s}}\end{array}$$

First, we measured ${\rho }_{aq}$ and ${X}_{s}$ for a 100 g/l NaCl brine. Then, a Berea sandstone core-sample with known initial permeability, was saturated with the brine and about 300 PV of supercritical CO_2_ was flooded through the core at a rate of 5 ml/min. After every 50 PV of CO_2_ injections, the core was inspected by scanning to determine ${l}_{d}$ after which permeability was measured. We then calculated $\beta$ for each ${l}_{d}$ and plotted the data. An initial ${S}_{s}$ correlation similar to Eq. (18) by replacing ${\overline{S} }_{g,d}$ with ${l}_{d}$ without the matching parameters was used to compute $\beta$. The saturation correlation ${S}_{s}$ was then optimized to fit the initial experimental data. The optimum correlation in Eq. (19) was then used throughout the various experiments. A brine concentration outside the range used in the main experimental set was used to calibrate the correlation and to ensure repeatability.

Equation (19) is used to estimate ${S}_{s}$ in the dry-out zone. Once ${S}_{s}$ is known, ${\Delta r}_{i}$ are computed from Eq. (17) and then $\beta$ for each ${l}_{d}$ calculated from Eq. (4).

## Discussion of results

### The mechanism of external salt precipitation

The mechanisms of salt precipitation can be grouped into two: salt cake formation at the injection inlet and drying effects. Under some favorable conditions, salt cake may form on the surface of the core inlet during early stages of brine vaporization before the onset of drying.

**Figure 4:**
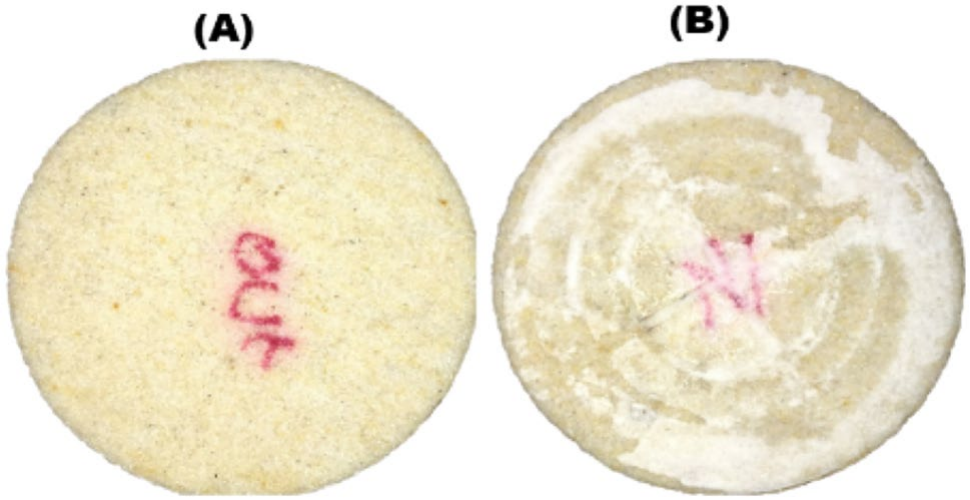
Photographs of Bentheirmer core after CO_2_ was injected at 1 mL/min into the core initially saturated with 120 g/L NaCl brine. (**A**) No salt cake observed at the core outlet. (**B**) Massive salt cake found at the injection inlet.

To investigate mechanisms of salt cake formation, a clean Bentheimer core was vacuum-saturated with 120 g/L NaCl brine and flooded with about 50 PV of dry supercritical CO_2_ at a rate of 1 mL/min. The Bentheimer core was selected because of its relatively high permeability. Pressure drop profiles were recorded during the CO_2_ flooding. From Fig. 4A, it can be observed that no salt was formed at the core outlet. Figure 4B shows massive salt cake deposition at the core inlet. At the onset of injection, when the core is fully saturated with brine, the injected supercritical CO_2_ leaves brine behind the inlet due to poor sweep of the brine by the injected fluid. Salinity of the brine increases gradually due to mass transfer of water from the brine into the dry supercritical CO_2_. If the initial brine salinity is high enough, the brine left behind at the inlet could reach supersaturation and precipitate salt onto the inlet surface. The precipitated salt creates a saturation gradient that draws more brine into the inlet region through capillary backflow, leading to precipitation of more salts at the inlet. Thus, the salt cake formation could increase with brine salinity and inefficient brine displacement at the injection inlet.

**Figure 5:**
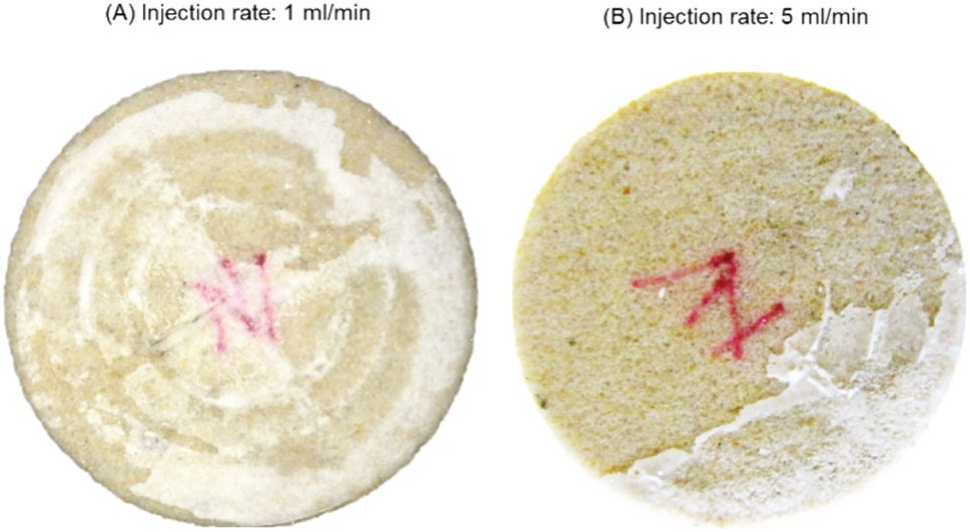
Photographs showing salt cake development at the core inlet when supercritical CO_2_ injection rate was increased from (**A**) 1 mL/min to (**B**) 5 mL/min. Increase in CO_2_ injection rate decreased the amount of deposited salt.

To investigate the impact of sweep on salt cake development, supercritical CO_2_ injection flow rate was increased from 1 to 5 mL/min. Salt deposition at the injection inlet decreased when the injection flow rate was increased from 1 to 5 mL/min (Fig. 5). The sweep at the injection inlet improved with increasing injection flow rate, leaving less brine behind the injection inlet for salt precipitation.

**Figure 6:**
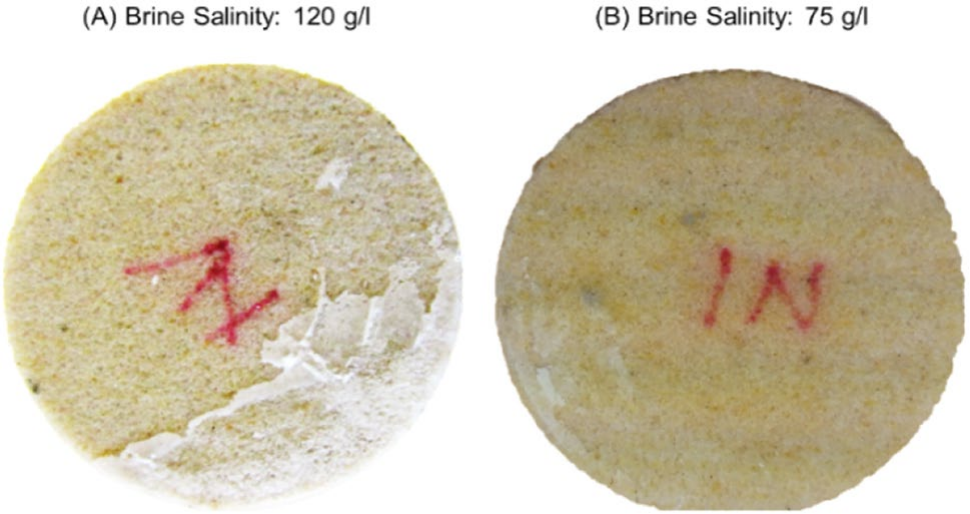
Photographs showing salt cake development at the core inlet when brine salinity was decreased from (**A**) 120 g/L to (**B**) 75 g/L. Decrease in brine salinity further decreased salt deposition.

The initial brine salinity was then reduced to 75 g/L, keeping the CO_2_ injection flow rate constant at 5 mL/min. The amount of salt cake precipitated at the injection inlet further decreased significantly when brine salinity was decreased (Fig. 6). At constant rate of vaporization, lowering the saturating brine salinity delays supersaturation, allowing a significant portion of the brine left behind at the injection inlet to be swept into the core which in tend reduces the amount of salt cake formed at the inlet.

From these experimental studies, it can be observed that salt cake deposition at the injection inlet during CO_2_ injection into saline porous media may depend on saturating brine salinity and the brine sweep at the injection inlet.

### Drying, extension of the dry-out region and injectivity impairment

**Figure 7:**
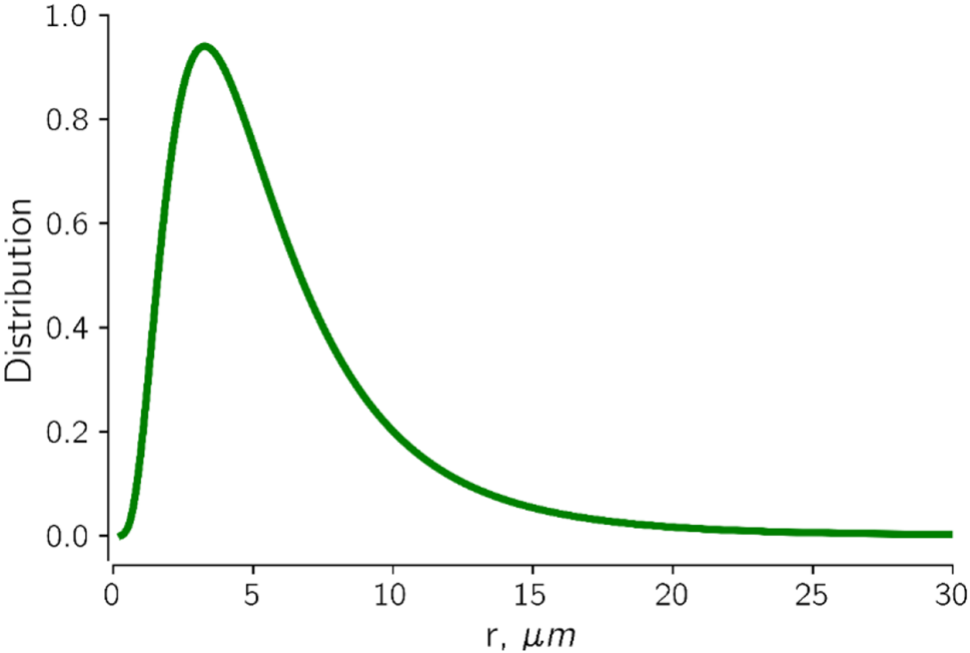
Distribution of tube radii representing pore size distribution of Berea sandstone core. The tube radii were drawn from a lognormal distribution with average tube radius of about 6 μm which is the average pore throat size of Berea sandstone.

Pore size distribution is required to model the porous medium with the *bundle-of-tubes* model derived in section "Modelling work". Based on a pore size distribution analysis from mercury injection on a Berea sandstone, Shi et al.^57^ have found an average pore radius of about 6.7 μm. Dullien and Dhawan^58^ reported pore constriction sizes between 0.5 and 5.0 μm and pore chamber sizes ranging from 5.0 to 50 μm in Berea sandstone. From these data, we calibrated our model to an average pore size of 6 μm using a lognormal distribution of tube radii, as shown in Fig. 7. In Fig. 7, it can be observed that tubes with radii greater than 20 μm consist of less than 5% of the total number of capillary tubes. The minimum tube radius was set to 0.38 μm.

Natural reservoir rocks have open pore bodies connected by two or more pore throats depending on the coordination number of the type of rock. The average coordination number of Berea sandstones is between 4 and 8^59,60^. The pore size distribution in Fig. 7 compares favorably with this range of coordination number.

**Figure 8:**
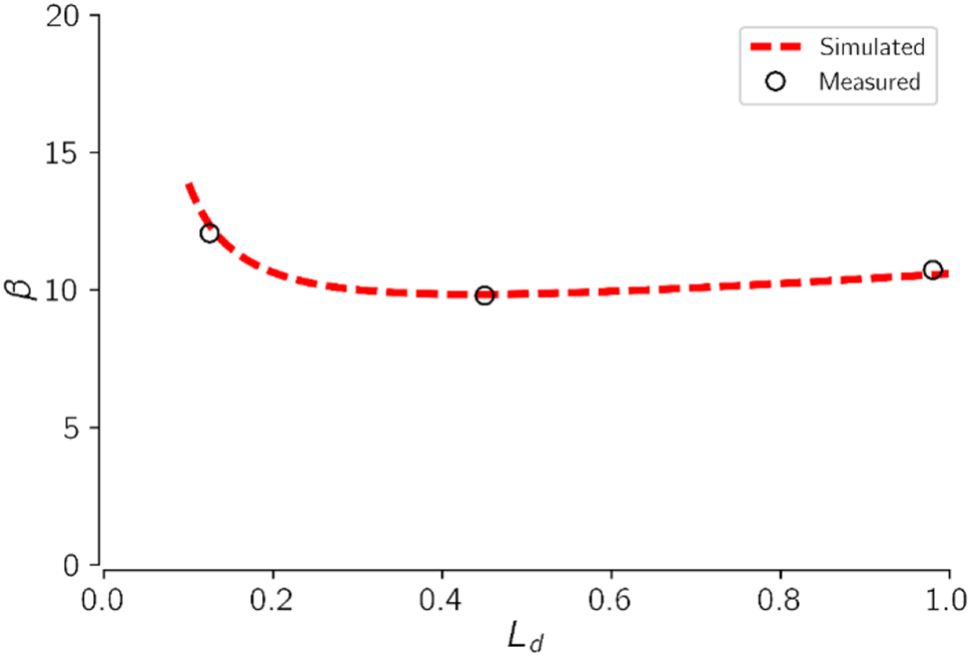
Effect of the advancing dry-out region on CO_2_ injectivity impairment. The core was initially saturated with LS brine. CO_2_ Injectivity change, and extent of the dry-out region were measured after every 100 PV of CO_2_ injection.

A Berea sandstone core with known initial permeability was saturated with LS brine prior to the drying experiment. The core was then flooded with supercritical CO_2_ at 5 ml/min. After every 100 PV of CO_2_ injection, the core was taken out and inspected, and the advancing dry-out region was measured to estimate ${l}_{d}$. CO_2_ injectivity change caused by salt precipitation was also measured and $\beta$ was computed. Figure 8 shows the impact of the advancing dry-out region, ${l}_{d}$ on CO_2_ injectivity impairment, $\beta$ for the experimental and modelling studies.

Figure 8 shows the simulation data agrees favorably with the measured data. It can be observed that CO_2_ injectivity impairment was highest at the onset of drying. Injectivity impairment decreased to a minimum for ${l}_{d}$ of about 0.45 and then increased slightly towards the core effluent end. At the commencement of drying, injectivity impairment could be induced by salt precipitation and relative permeability effects. Brine vaporization rate is highest close to the inlet region because of the high capillary driven back-fluxes. When the brine attains supersaturation, salt could be precipitated into the pores which in turn could reduce injectivity. Also, at the onset of drying, a large portion of the rock still contains brine. Capillary backflow may also drive the aqueous phase towards the injection inlet. The presence of mobile brine in the pores, could reduce the space available for CO_2_ flow, thus decreasing the relative permeability of CO_2_, which could also have an impact on injectivity around the inlet region.

As the drying front advances into the core, brine vaporization and salt precipitation are expected to decrease as a result of the reduced fluxes. In addition, the mobile brine saturation will decrease as more brine is swept out of the core. This will increase the relative permeability to CO_2_. When the core is almost fully dried, brine vaporization and salt precipitation at the outlet end of the core are almost negligible. Also, the absence of mobile brine in the rock further improves the relative permeability of the rock to CO_2_. However, the capillary driven back fluxes and changes in the distribution of deposited salt as a result of the continuous injection of large pore volumes of CO_2_ after salt deposition was probably responsible for the slight increase in injectivity impairment towards the end of the rock. Salt redistribution within the pores can cause heterogeneous salt deposition which tends to impair CO_2_ injectivity as also observed by other researchers^14,38,61^.

### Effect of brine salinity

**Figure 9:**
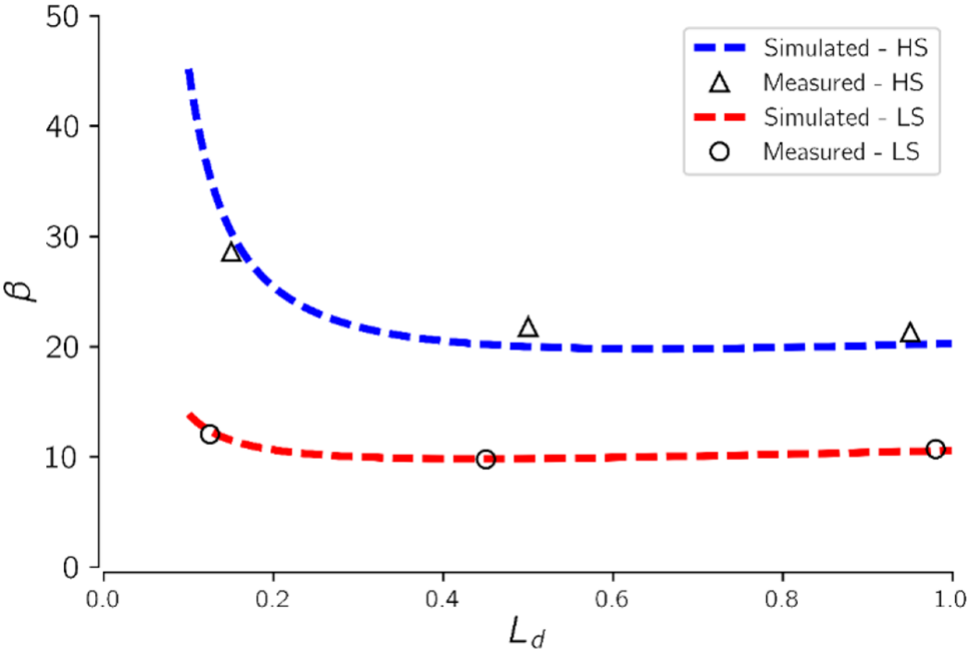
Effect of brine salinity on CO_2_ injectivity in relation to advancement of the dry-out region. The magnitude of CO_2_ injectivity impairment is seen to increase more than two-fold when brine salinity was doubled from 75 to 150 g/l.

To investigate the effect of brine salinity, the experiment was repeated with HS brine. The measured and simulated results for the LS and HS brine cases are presented in Fig. 9.

Generally, CO_2_ injectivity impairment increased more than two-fold when brine salinity was doubled from 75 to 150 g/l (Fig. 9). This result compares well with the findings of Jeddizahed and Rostami^62^ who reported increased permeability impairment from 21 to 50%, when brine salinity was increased from 50 to 100 g/l, under similar experimental conditions. Other researchers^8,25,27^ have also reported direct increase in CO_2_ injectivity impairment proportional to increase in brine salinity.

Figure 9 also suggests that the same mechanisms are responsible for CO_2_ injectivity impairment as the dry-out region extents from the injection inlet regardless of the initial saturating brine salinity. Increase in brine salinity from LS to HS, increased the magnitude of CO_2_ injectivity impairment but had no direct impact on the development of CO_2_ injectivity impairment during the drying process. Also, the simulation results compare favorably with the measured data for the increased brine salinity.

Generally, solid salt saturation increases with increase in brine salinity^10,20,26,44^. However, it can be observed that increase in brine salinity had negligible effect on the mechanisms underlying the changes in CO_2_ injectivity as the dry-out zone extends into the rock. The two main mechanisms, rate of salt precipitation during the drying process and relative permeability effects are for the most part dependent on CO_2_ injection rate. Increase in brine salinity only increases the magnitude of salt precipitated but the rate of precipitation depends primarily on the brine vaporization rate. Also, the relative permeability effects are influenced by the amount of mobile brine present in the rock in real time.

## Conclusion

In terms of storage space, deep saline formations are the most viable candidates for CCUS. However, salt precipitation during CO_2_ injection into deep saline reservoirs could impair CO_2_ injectivity and reduce storage potential. Insight into the drying process, the changes in the dry-out region and its impact on CO_2_ injectivity could improve understanding of the mechanisms of drying and salt precipitation in saline reservoirs. Core-flood experiments were conducted to investigate the mechanisms of drying during CO_2_ injection into saline reservoirs and the impact of the size of the advancing dry-out region on CO_2_ injectivity. The experimental results were used to calibrate a *bundle-of-tubes* model to track the size of the dry-out region and its impact on CO_2_ injectivity impairment induced by solid salt precipitation. Some highlights of our findings include the following:

1. Salt could be precipitated externally on the surface of the injection inlet in the form of filter a cake. Brine salinity and sweep efficiency of brine around the injection inlet have been identified as the main underlying parameters of external salt precipitation.
2. For drying and internal salt precipitation, CO_2_ injectivity impairment was highest at the onset of the drying process due to high brine vaporization rate and low relative permeability of CO_2_. Injectivity improved slightly as the drying front advanced into the middle of the core but decreased gradually towards the end of the drying process. Overall, increase in the dry-out region did not impose significant effect on the magnitude of CO_2_ injectivity.
3. Doubling the initial brine salinity from 75 to 150 g/l reduced CO_2_ injectivity more than two-fold, but the successive change in injectivity due to the extension of the dry-out region was independent of initial brine salinity.

Although the analytical model was largely basic, the accompanying experimental findings are very important for understanding the mechanisms of drying and salt precipitation in saline reservoirs. Insight from the current study could provide a solid basis for understanding the relationship between CO_2_ injectivity induced by salt deposition and the advancement of a dry-out region.

## Data Availability

The datasets used and/or analyzed during the current study are available from the corresponding author on reasonable request.
